# Monitorização Residencial da Pressão Arterial e Controle Pressórico em Hipertensos Tratados

**DOI:** 10.36660/abc.20220038

**Published:** 2022-07-29

**Authors:** Andréa Araujo Brandão, Weimar Kunz Sebba Barroso, Audes Feitosa, Eduardo Costa Duarte Barbosa, Roberto Dischinger Miranda, Priscila Valverde de Oliveira Vitorino, Roberto Pozzan, Lucio Paulo Ribeiro, Abraham Epelman, Giovanni Alves Saraiva, Fabio Serra Silveira, Antônio Almeida Braga, Marco Mota Gomes

**Affiliations:** 1 Universidade do Estado do Rio de Janeiro Rio de Janeiro RJ Brasil Universidade do Estado do Rio de Janeiro, Rio de Janeiro, RJ – Brasil; 2 Universidade Federal de Goiás Goiânia GO Brasil Universidade Federal de Goiás, Goiânia, GO – Brasil; 3 Universidade de Pernambuco Recife PE Brasil Universidade de Pernambuco, Recife, PE – Brasil; 4 Santa Casa de Misericórdia de Porto Alegre Porto Alegre RS Brasil Santa Casa de Misericórdia de Porto Alegre, Porto Alegre, RS – Brasil; 5 Universidade Federal de São Paulo São Paulo SP Brasil Universidade Federal de São Paulo, São Paulo, SP – Brasil; 6 Hospital Israelita Albert Einstein São Paulo SP Brasil Hospital Israelita Albert Einstein, São Paulo, SP – Brasil; 7 Pontifícia Universidade Católica de Goiás Goiânia GO Brasil Pontifícia Universidade Católica de Goiás, Goiânia, GO – Brasil; 8 Servier do Brasil Rio de Janeiro RJ Brasil Servier do Brasil, Rio de Janeiro, RJ – Brasil; 9 Prevencor Recife PE Brasil Prevencor, Recife, PE – Brasil; 10 Imedi Recife PE Brasil Imedi, Recife, PE – Brasil; 11 Icordis Recife PE Brasil Icordis, Recife, PE – Brasil; 12 Centro de Pesquisa Clínica do Coração Aracaju SE Brasil Centro de Pesquisa Clínica do Coração, Aracaju, SE – Brasil; 13 Procape Recife PE Brasil Procape / MCor, Recife, PE – Brasil; 14 Centro Universitario CESMAC Maceió AL Brasil Centro Universitario CESMAC, Maceió, AL – Brasil

**Keywords:** Hipertensão, Controle, Fatores de Risco, Pressão Arterial, Monitores de Pressão Arterial/métodos, Telemonitoramento, Telemedicina

## Introdução

As taxas de controle da pressão arterial (PA) são muito baixas no Brasil e no mundo, cerca de 20%.^1-3^ Hipertensos tratados e não controlados mantêm elevado risco de eventos cardiovasculares (CV) e de mortalidade, assemelhando-se aos indivíduos não tratados.^4^

De acordo com recomendações recentes,^1,2,5,6^ o controle pressórico deve ser verificado pela PA de consultório (PAC), e pela medida fora do consultório. Assim, é possível caracterizar os diferentes fenótipos da hipertensão arterial,^1,2,5,6^ importante na determinação do prognóstico e da terapia individualizada.^1,2,5-7^

A Monitorização Residencial da PA (MRPA) é o registro realizado pelo paciente ou outra pessoa treinada utilizando um aparelho automático, por vários dias, durante a vigília, no seu domicílio, com protocolo determinado. Tem boa aceitação pelo paciente, custo baixo, boa reprodutibilidade e valor prognóstico.^5-7^ A MRPA associa-se a menor inércia terapêutica e ao maior engajamento e adesão do paciente ao tratamento, especialmente quando combinada com educação e aconselhamento,^1,2,6^ contribuindo para maior proteção CV.^5,6,8^

O presente artigo teve como objetivo comparar as taxas de controle da PA pela medida no consultório e pela MRPA em duas populações de hipertensos em tratamento. Os indivíduos foram avaliados em 2019 e em 2020, após a introdução da MRPA de forma mais regular e frequente na prática de 274 consultórios médicos de cinco regiões brasileiras.

## Métodos

Trata-se de um estudo multicêntrico, de dois cortes transversais, parte do registro nacional do controle da hipertensão arterial avaliado pela medida da PAC e MRPA (Registro LHAR).

A PAC considerada foi a média de duas medidas realizadas com aparelho oscilométrico validado da marca OMRON modelo HEM-7320 no primeiro dia do protocolo de MRPA. O mesmo equipamento foi utilizado para a MRPA. Os pacientes ou seus acompanhantes foram instruídos a realizar seis medidas diárias de PA.^5^ Os exames foram analisados por meio da plataforma TeleMRPA (www.telemrpa.com), ferramenta de laudo à distância por telemonitoramento.

Dois pontos de corte foram considerados para determinar o controle da PAC: < 140/90mmHg e < 130/80mmHg. Para a MRPA, o ponto de corte foi < 130/80mmHg.^1^ Embora esses valores sejam menores que os adotados pelos europeus,^2,6^ mostraram maior correlação com PAC de 140/90mmHg e associaram-se a menor risco de lesão de órgão-alvo, de desfecho CV e mortalidade.^9^ As taxas de controle foram analisadas por sexo, grupo etário (≥ 60 anos e < 60 anos) e classificação do Índice de Massa Corporal (IMC).

A frequência dos fenótipos da HA foi determinada nos anos de 2019 e 2020, considerando-se PAC normal <140 e 90mmHg e MRPA normal <130 e 80mmHg ^1,5,6^ Seguem os fenótipos: 1) hipertensão controlada (HC): PAC e MRPA normais; 2) hipertensão do avental branco não controlada (HABNC): PAC anormal e MRPA normal; 3) hipertensão mascarada não controlada (HMNC): PAC normal e MRPA anormal; e 4) hipertensão não controlada (HNC): PAC e MRPA anormais.

Todos os participantes leram e assinaram o termo de consentimento livre e esclarecido. O estudo foi aprovado pelo Comitê de Ética em Pesquisa da Universidade Federal de Goiás (CAAE 08208619.8.0000.5078). Os dados foram analisados pelo do programa SPSS 27.0 (SPSS Inc.), considerando 5% como nível de rejeição da hipótese de nulidade para o Teste “t” de Student e Teste do Qui-quadrado.

## Resultados e discussão

Foram incluídos 5324 indivíduos, sendo 2538 avaliados em 2019 e 2786 em 2020. A maioria da amostra foi composta por mulheres (62,2%), o que é frequentemente observado em estudos clínicos no Brasil,^10,11^ e provavelmente refletem um maior cuidado da mulher com sua saúde.^3,12^ A amostra de 2020 tinha média de idade significativamente maior que o grupo de 2019. As médias de pressão arterial sistólica (PAS) e pressão arterial diastólica (PAD) foram menores na MRPA que no consultório, -6,6/-4,5mmHg, respectivamente, em acordo com outras publicações.^1,5,6,10^ Foram observadas médias da PAD no consultório e na MRPA menores em 2020 comparadas a 2019, porém a diferença observada entre os dois grupos foi menor que 1mmHg (Tabela 1). Em 47,7% dos casos, não houve registro da medicação anti-hipertensiva.

**Tabela 1:** Características demográficas, índice de massa corporal e medidas de pressão arterial das amostras de pacientes de 2019 e 2020

Variável	Total (n=5324)	2019 (n=2538)	2020 (n=2786)	Teste estatístico	Valor de p
Sexo (M/F) (%)	37,8/62,2	38,1/61,9	37,5/62,5	χ^2^=0,193	0,671
Idade (anos)	61,66±14,9	59,72±15,1	63,43±14,5	t=9,085	<0,0001
Idosos (≥ 60 anos) (%)	58,1	52,7	63,1	χ^2^=58,825	<0,0001
Índice de massa corporal (kg/m^2^)	28,6±5,2	28,6±5,1	28,7±5,3	t=0,804	0,421
Sobrepeso/Obesidade (%)	41,3/34,4	42,2/33,8	40,4/35,1	χ^2^=1,663	0,435
PAS consultório (mmHg)	132,2±19,8	132,4±19,4	132,1±20,2	t=0,610	0,542
PAD consultório (mmHg)	82,5±11,9	82,7±12,0	82,1±11,8	t=2,373	<0,02
PAS MRPA (mmHg)	125,6±15,9	125,9±16,1	125,4±15,7	t=1,208	0,227
PAD MRPA (mmHg)	77,9±9,5	78,6±9,3	77,3±9,6	t=4,823	<0,0001

PAS: pressão arterial sistólica; PAD: pressão arterial diastólica; MRPA: monitorização residencial da pressão arterial. Teste t e qui-quadrado.

**Figura 1:**
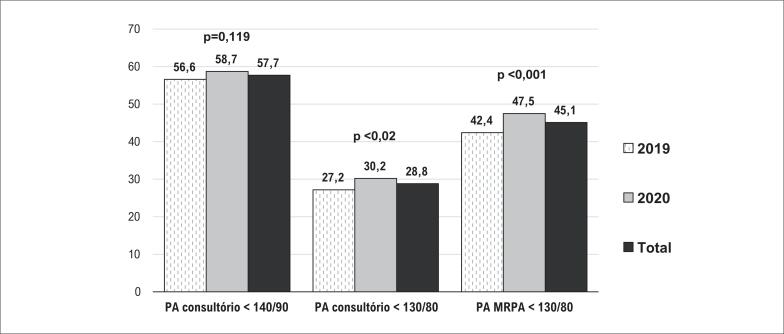
Taxas de alcance da meta pressórica pela pressão arterial de consultório e pela MRPA em 2019 e 2020. PA: pressão arterial; MRPA: monitorização residencial da pressão arterial. Teste Qui-quadrado.

As taxas de controle da PA na amostra total foram 57,7% pela PAC < 140/90mmHg, 28,8% pela PAC < 130/80mmHg e 45,1% pela MRPA < 130/80mmHg. (Figura 1). As taxas de controle da PAC com a meta padrão (<140/90mmHg) foram maiores (57,7%) do que o registrado no Brasil e em outros países,^1,3^ contudo já observadas em outros estudos brasileiros quando hipertensos são tratados por médicos especialistas, em especial cardiologistas.^10,11^

Em comparação a 2019, em 2020, houve aumento das taxas de controle para PAC < 130/80mmHg (27,2% vs. 30,2%; p<0,02) e MRPA < 130/80mmHg (42,4% vs. 47,5%; p<0,0001) (Figura 1). O estudo SPRINT^13^ demonstrou maior proteção CV com o alcance de metas mais rigorosas da PA, o que tem sido considerado pelas diretrizes.

Importante ressaltar que a pandemia por COVID-19 ocorreu a partir de 2020 e poderia ter impactado negativamente as taxas de controle da PA; entretanto, houve aumento das taxas de controle. Estudo brasileiro recente com mais de 50 000 indivíduos avaliados não encontrou influência da pandemia sobre as taxas de controle pela PAC ou pela MRPA.^14^

Os idosos, em geral com maior dificuldade para o controle da PA,^1,2,12^ mostraram aumento das taxas de controle pela PAC <130/80mmHg e pela MRPA. Estudos com hipertensos idosos têm enfatizado os benefícios de reduções mais intensas da PA na proteção CV.^15,16^ Nos obesos, condição de grande impacto sobre os valores de PA,^1,2^ foram observados aumentos das taxas de controle da PA de 2019 para 2020, por todos os critérios empregados. Estes dados reforçam a importância da avaliação da PA pelos dois métodos.^1,2,5,6^

**Figura 2:**
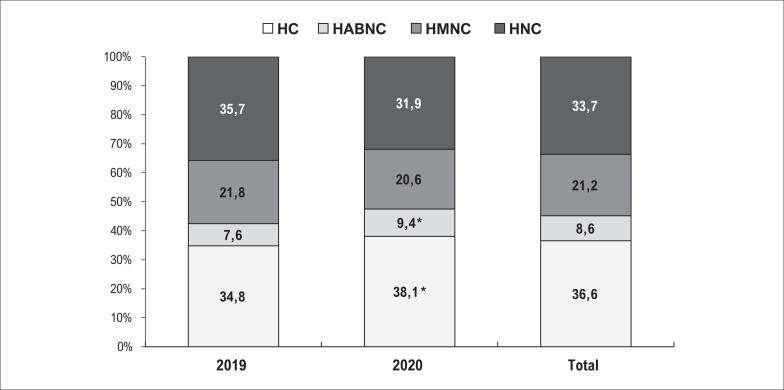
Distribuição dos fenótipos de pressão arterial em 2019 e 2020. HC: hipertensão controlada; HABNC: hipertensão do avental branco nãocontrolada; HMNC: hipertensão mascarada não-controlada; HNC: hipertensão não-controlada. Teste qui-quadrado. *p < 0,05.

Na amostra total, a distribuição dos fenótipos de hipertensão mudou significativamente de 2019 para 2020, com aumento das taxas de HC e HABNC e redução de HMNC e HNC (Figura 2). Assim, houve uma melhora na distribuição percentual dos fenótipos de um ano para o outro, mesmo usando pontos de corte mais rigorosos para a MRPA. Além disso, a distribuição dos fenótipos revelou maiores taxas de HMNC e menores de HABNC do que o estimado pelas Diretrizes Brasileiras de Hipertensão Arterial 2020^1^ e o encontrado em estudo brasileiro com mais de 6500 pacientes,^10^ o que pode ser explicado pela utilização de ponto de corte menor para a MRPA.^17,18^

Destacam-se algumas limitações do estudo: 1) a análise de dois cortes transversais de pacientes hipertensos, o que não permite análises da evolução do tratamento; 2) não são conhecidos dados clínicos mais detalhados como estágio da hipertensão arterial, presença de comorbidades e outros fatores de risco CV; 3) não foi possível a análise do uso de medicamentos, informação registrada em menos da metade dos pacientes e de forma incompleta. Por outro lado, destaca-se o número expressivo de pacientes avaliados, com amostras em 2019 e 2020 relativamente homogêneas para a maioria das características demográficas e clínicas consideradas.

Em conclusão, os dados avaliados revelaram aumento da taxa de controle da PA pela PAC <130/80mmHg e pela MRPA em hipertensos tratados. Em 2019, foi iniciado o uso da MRPA de forma mais regular e frequente nesses consultórios, o que pode ter influenciado a prática clínica dos médicos, por uma maior atenção na avaliação da PA fora do consultório e consequente aumento nas taxas de controle da PA de 2019 para 2020. Ressalta-se também que a MRPA facilita o maior engajamento do paciente ao seu tratamento, associa-se à maior adesão e melhor controle da PA.^19,20^ Esses fatores em conjunto mostram importante contribuição da MRPA para aumentar as taxas de controle da PA.

## *Material suplementar

Para informação adicional, por favor, clique aqui


