# Supplementary material for: Monitorização Residencial da Pressão Arterial e Controle Pressórico em Hipertensos Tratados
Source: Arq Bras Cardiol. 2022 Jul 29;119(2):353–7. [Article in Portuguese] doi: 10.36660/abc.20220038 (PMC9363073; doi:10.36660/abc.20220038)
Supplement: Supplementary file 1 [file 2022-0038_CC_LHAR_Supplemental_file_coinvestigators_list_18.01.22.pdf]

Coinvestigadores nacionais

Ademar Martins da Silva (Mamanguape - PB), Admardo Guimarães Cassar (Rio de Janeiro, RJ), Adriano Renato Fonseca Boiça (Caieiras - SP), Airton Minotto (Caxias do Sul – RS), Alcirley de Almeida Luiz (Cascavel - PR), Alexandra Cunha Marinho (Nova Iguaçu - RJ), Alexandre Konovaloff Jannotti (Belo Horizonte - MG), Almir Nóbrega da Silva Filho (João Pessoa – PB), Ana Paula Battistuzzi (Indaiatuba – SP), Anderson Carlos De Almeida (Caruaru - PE), André K Vidigal (Caruaru - PE), Andre Leonardo F. de Moura (Ribeirão Preto – SP), Andre Luiz Gabrieli (Caxias do Sul - RS), Anibal Prata Barbosa (Duque de Caxias - RJ), Antonio Almeida Braga (Recife - PE), Aristeu Haroldo Kimio Mizuta (São José dos Campos - SP), Armando Elias Chamma (Botucatu – SP), Breno Gontijo de Camargos (Brasília - DF), Bruno Augusto Alcova Nogueira (São José dos Campos - SP), Bruno Daniel Ferrari (Assis - SP), Carlaile Antonio Sarmento de Araujo Costa (Teresina - PI), Carlos Alberto Misson Ferreira Filho (Votuporanga - SP), Carlos Alberto Ramos Macias (Maceió - AL), Carlos Alberto Santiago Nunes (Canoas - RS), Carlos Efrem Lustosa da Costa (Fortaleza - CE), Carlos Filinto de Almeida (Campo Grande - MS), Carlos Henrique de Moraes Costa (Campo Limpo Paulista - SP), Celso Cesar Carneiro (Florianópolis - SC), Cesar Ricardo Soares Medeiros (Ribeirão Preto - SP), Cicero Bittar Branco (Campos dos Goytacazes - RJ), Claudinelli Alvarenga Aguilar (Goiânia - Goiás), Cristiano de Melo Rangel Gomes (Saquarema - RJ), Cristiano Duarte da Silveira (Avaré - SP), Cristiano Ventura (Gravataí - RS), Daniel Siqueira Barbosa (Teresina - PI), Daniella Rosano Gregorini (Jundiaí - SP), Diego Junier Roumow (Santa Maria – RS), Edinaldo Jorge Piedade Malheiros (Osasco - SP), Edison Ramos Migowski de Carvalho (Rio de Janeiro - RJ), Edmirton Soares de Macedo (Teresina - PI), Edson Ferreira (Gravataí – RS), Eduardo Erico Zen (Curitiba - PR), Eduardo Messias de Carvalho Teixeira (Patrocínio - MG), Edson Rossi (Espírito Santo do Pinhal – SP), Elizabeth da Rosa Duarte (Porto Alegre - RS), Elizabeth do Espirito Santo Cestário (Votuporanga - SP), Ernesto Macedo Junior (São Paulo – SP), Érika Maria Gonçalves Campana (Niterói- RJ), Erwin Soliva Júnior (Cascavel – PR), Evanio Rodrigues Cordeiro (Montes Claros - MG), Fabiano Machado Pêgas (Governador Valadares - MG), Fabio Alban (Caxias do Sul - RS), Fabio Calil Vieira (Cabo Frio - RJ), Fabio Augusto Ribeiro Furlan (Caçapava - SP), Fabio Serra Silveira (Aracaju - SE), Fabricio Rodrigues Dos Anjos (Pouso Alegre - MG), Fernando Augusto Alves da Costa (São Paulo – SP), Fernando Patricio Aliaga Mora (São Paulo - SP), Fernanda Cristina Teixeira Rodrigues (Nova Iguaçu - RJ), Fernando Albuerne Bezerra (Mossoró - RN), Fernando Alfredo da Fonseca (Petrópolis - RJ), , Fernando Jorge Vallada Roselino (Ribeirão Preto - SP), Fernando Lucas Dipe Prates Miranda (Poços de Caldas - MG), Flavia Karina Silva e Oliveira (São José dos Campos - SP), Flávia Moretzsohn Silva Oliveira Rocha (Ubá - MG), Geraldo de Souza Neto (Barretos – SP), Giovanni Alves Saraiva (Recife - PE), Gisele Alves Balloussier (Rio de Janeiro - RJ), Gustavo Paz Esteves Ferreira Fonseca (Goiânia-GO), Hailton Boing Jr (Brusque - SC) Hiran De Paula Haun (Itabaiana - SE), Hueverson Junqueira Neves (Araguaína - TO), Humberto Cesar Tinoco (Itaboraí - RJ), Idalia de Sousa Andrade (São Paulo - SP), Ivanildo Palmeira (Recife - PE), João Felix de Morais Filho (Natal - RN), João Francisco Martins Pacheco (Belém - PA), João Miguel Malta Dantas (Colatina - ES), Joao Vicente Marques de Oliveira (São João da Boa Vista - SP), Jonathan Scapin Zagatti (Jales - SP), Jorge Luiz Scribel da Silva (Canoas - RS), Jose Albuquerque De Figueiredo Neto (São Luís - MA), Jose Cássio de Abreu (Jacareí - SP), Jose de Arimatea Barbosa da Silva (Campina Grande - PB), Jose Luiz Lima Mascarenhas (Rio de Janeiro - RJ), Jose Maria Cotta Junior (Manaus - AM), Jose Miguel Gonçalves (João Pessoa - PB), Juan Manuel Sanchez Arana (Santo André - SP), Katia Barbosa de Barros (São Paulo – SP), Lilian Barbosa Tourinho Batista (Balneário

Camboriú - SC), Lucia Cristina Figueiredo Lenzi (Rio de Janeiro - RJ), Luis Eduardo Targino Dias (São Mateus - ES), Luiza Maria Figueiredo dos Santos (Rio de Janeiro - RJ), Marcio Andre Ferreira Silva (Feira de Santana - BA), Marco Antonio Mussato da Silva (Paranavaí – PR), Marco Aurélio Borges (Presidente Prudente – SP)Marcus Antonio Lemos de Barros (Aracaju - SE), Maria Christina Cavalcanti Ballut (Manaus - AM), Maria do Socorro Rodrigues Oliveira de Araujo (João Pessoa - PB), Marta Souza Figueiredo (Feira de Santana - BA), Miguel Luiz Cortez (Praia Grande – SP), Miguel Pereira Ribeiro (Campina Grande - PB), Newton Cesar Carrinhena (São Paulo - SP), Olavo Raimundo Guimarães Junior (Alfenas - MG), Osmar Araujo Calil (Vitória - ES), Patricia Ayd de Oliveira Bittencourt (Macaé - RJ), Paulo Eduardo Ballve Behr (Porto Alegre - RS), Rafael Lois Greco (São José do Rio Preto – SP), Rafael Moreira Lima (Cabo Frio - RJ), Regina Lúcia Moysés (Brasília - DF), Renata Novis dos Santos (São Paulo - SP), Renato Frange Miziara Mussi (Fernandópolis - SP), Ricardo Marques Beato (Bebedouro - SP), Roberto Lebet (Ribeirão Preto - SP), Rodolfo Roberto Andrade Sciampaglia (Sumaré - SP), Rodrigo Lopes de Sousa (Natal - RN), Rodrigo Morato Valério (Florianópolis - SC), Rodrigo Palavro (Florianópolis - SC), Rodrigo Portela de Santana (Campinas – SP), Rogerio Carlessi (Criciúma - SC), Rosana Cruz (Caxias do Sul - RS), Rosângela Leocádio (Recife - PE), Sandro Toledo (Cascavel – PR), Sebastião Gomes de Almeida (Brejo Santo - CE), Sérgio Ricardo Duarte Moutinho (São Gonçalo - RJ), Sidney Araujo São Jose (Belo Horizonte - MG), Sonia Regina Magalhães Garcia (Jaú - SP), Tania Velka Omena Monte De Almeida (Maceió - AL), Tarciso Oliveira Bomfim (Ipatinga - MG), Valdir do Nascimento (São Paulo - SP), Vilma Helena Marchon Leao Burlamaqui (Niterói - RJ), Wagner Ramos da Silva (Fortaleza - CE), Walmir de Vasconcelos Ratier Thomaz (Rio de Janeiro - RJ), Wenderson Tavares dos Santos (Belo Horizonte).
