# Supplementary material for: Monitorização Residencial da Pressão Arterial e Controle Pressórico em Hipertensos Tratados
Source: Arq Bras Cardiol. 2022 Jul 29;119(2):353–7. [Article in Portuguese] doi: 10.36660/abc.20220038 (PMC9363073; doi:10.36660/abc.20220038)
Supplement: Translation

# Translation: Home Blood Pressure Monitoring and Blood Pressure Control in Treated Hypertensives

**Authors:** Andréa Araujo Brandão, Weimar Kunz Sebba Barroso, Audes Feitosa, Eduardo Costa Duarte Barbosa, Roberto Dischinger Miranda, Priscila Valverde de Oliveira Vitorino, Roberto Pozzan, Lucio Paulo Ribeiro, Abraham Epelman, Giovanni Alves Saraiva, Fabio Serra Silveira, Antônio Almeida Braga, Marco Mota Gomes

**Affiliations:** 1 Rio de Janeiro RJ Brazil Universidade do Estado do Rio de Janeiro, Rio de Janeiro, RJ – Brazil; 2 Goiânia GO Brazil Universidade Federal de Goiás, Goiânia, GO – Brazil; 3 Recife PE Brazil Universidade de Pernambuco, Recife, PE – Brazil; 4 Porto Alegre RS Brazil Santa Casa de Misericórdia de Porto Alegre, Porto Alegre, RS – Brazil; 5 São Paulo SP Brazil Universidade Federal de São Paulo, São Paulo, SP – Brazil; 6 São Paulo SP Brazil Hospital Israelita Albert Einstein, São Paulo, SP – Brazil; 7 Goiânia GO Brazil Pontifícia Universidade Católica de Goiás, Goiânia, GO – Brazil; 8 Rio de Janeiro RJ Brazil Servier do Brasil, Rio de Janeiro, RJ – Brazil; 9 Recife PE Brazil Prevencor, Recife, PE – Brazil; 10 Recife PE Brazil Imedi, Recife, PE – Brazil; 11 Recife PE Brazil Icordis, Recife, PE – Brazil; 12 Aracaju SE Brazil Centro de Pesquisa Clínica do Coração, Aracaju, SE – Brazil; 13 Recife PE Brazil Procape / MCor, Recife, PE – Brazil; 14 Maceió AL Brazil Centro Universitario CESMAC, Maceió, AL – Brazil

**Keywords:** Hypertension, Control, Risk Factors, Arterial Pressure, Blood Pressure Monitors/methods, Telemonitoring, Telemedicine

## Introduction

Blood pressure (BP) control rates are very low in Brazil and in the world, around 20% only.^1-3^ Patients with uncontrolled hypertension despite treatment remain at high risk for cardiovascular (CV) events and mortality, comparably to untreated individuals.^4^

According to recent recommendations,^1,2,5,6^ BP control should be monitored by both office BP and out-of-office BP measurements. In this way, it is possible to determine different hypertension phenotypes,^1,2,5,6^ which is very important to determine the prognosis and individualized therapy.^1,2,5-7^

Home monitoring BP (HMBP) is the measurement of BP at home, performed by the patient or another trained person for several days, while awake, using an automated device and a pre-established protocol. HBPM has a low cost, good reproducibility, and good prognostic value. It is well accepted by patients^5-7^ and is associated with lower therapeutic inertia and higher patient engagement and treatment compliance, especially when combined with education and counseling,^1,2,6^ contributing to greater CV protection.^5,6,8^

The present study aimed to compare BP control rates between office BP and HBPM in two treated hypertensive populations. The individuals were assessed in 2019 and 2020, after the implementation of HBPM into the practice of 274 centers in five different regions of Brazil.

## Methods

This was a multicentric study, of two cross-sectional cohorts, part of the national registry of BP control, evaluated by office BP monitoring and HBPM.

Office BP was considered as the mean of two measurements performed using a validated oscillometric device (OMRON, model HEM-73,20) on the first day of the HBPM protocol. The same device was used for HBPM. Patients or their caregivers were instructed to take six BP measurements daily.^5^ The tests were analyzed through the TeleMRPA platform (www.telemrpa.com), an instrument that allows remote BP data analysis by telemonitoring.

For analysis of office BP control, two BP cutoffs were considered – < 140/90mmHg and < 130/80mmHg – and for HBPM, the cutoff of < 130/80mmHg was used.^1^ Although these values are lower than those adopted by Europeans,^2,6^ they showed a higher correlation with an office BP of 140/90mmHg and were associated with lower risk of target organ damage, of CV outcomes and mortality.^9^ The rates of BP control were analyzed by sex, age group (≥ 60 years and < 60 years), and body mass index (BMI) classification.

The frequency of hypertension phenotypes was determined in 2019 and 2020, considering a BP <140 and 90mmHg and <130 and 80mmHg as normal office BP and HBPM respectively.^1,5,6^ The phenotypes were classified as: 1) controlled hypertension (CH): normal office BP and HBPM; 2) white coat uncontrolled hypertension (WCUH): abnormal office BP and normal HBPM; 3) masked uncontrolled hypertension (MUH): normal office BP and abnormal HBPM; and 4) uncontrolled hypertension (UH): abnormal office BP and HBPM.

All patients read and signed the informed consent form. The study was approved by the ethics committee of the Federal University of Goias (CAAE 08208619.8.0000.5078). Data analysis was performed using the SPSS 27.0 (SPSS Inc.), by the Student’s t-test and the chi-square test, rejecting the null hypothesis at the 5% significance level.

## Results and discussion

A total of 5,324 individuals were included in the study, 2,538 in 2019 and 2,786 in 2020. Most patients were women (62;2%), as frequently observed in clinical studies conducted in Brazil,^10,11^ which probably reflects the fact that women care more about their health than men.^3,12^ Mean age of the 2020 cohort was significantly higher than the 2019 cohort. Mean systolic BP (SBP) and mean diastolic BP (DBP) were lower by HBPM than office BP (-6,6/-4,5mmHg, respectively), which is in accordance with previous studies.^1,5,6,10^ In addition, mean DBP measured in the office and by HBPM was lower in 2020 compared with 2019, although the difference between the groups was lower than 1 mmHg (Table 1). There was no record of antihypertensive medication use in 47.7% of the cases.

**Table 1:** Demographic characteristics, body mass index and blood pressure levels of the 2019 and 2020 cohorts

Variable	Total (n=5324)	2019 (n=2538)	2020 (n=2786)	Statistical test	p value
Sex (M/F) (%)	37.8/62.2	38.1/61.9	37.5/62.5	χ^2^=0.193	0.671
Age (years)	61.66±14.9	59.72±15.1	63.43±14.5	t=9.085	<0.0001
Elderly (≥ 60 years) (%)	58.1	52.7	63.1	χ^2^=58.825	<0.0001
Body mass index (Kg/m2)	28.6±5.2	28.6±5.1	28.7±5.3	t=0.804	0.421
Overweight/Obesity (%)	41.3/34.4	42.2/33.8	40.4/35.1	χ^2^=1.663	0.435
Office SBP (mmHg)	132.2±19.8	132.4±19.4	132.1±20.2	t=0.610	0.542
Office DBP (mmHg)	82.5±11.9	82.7±12.0	82.1±11.8	t=2.373	<0.02
HBPM SBP (mmHg)	125.6±15.9	125.9±16.1	125.4±15.7	t=1.208	0.227
HBPM DBP (mmHg)	77.9±9.5	78.6±9.3	77.3±9.6	t=4.823	<0.0001

SBP: systolic blood pressure; DBP: diastolic blood pressure; HBPM: home blood pressure monitoring; t-test and chi-square test.

Rates of BP control were 57.7% by using an office BP < 140/90mmHg, 28.8% using an office BP < 130/80mmHg and 45.1% pela MRPA < 130/80mmHg (Figure 1). When the standard target (< 140/90mmHg) was adopted, BP control rates were higher (57.7%) than those registered in Brazil and other countries,^1,3^ but similar to those reported in previous Brazilian studies when hypertensive patients were treated by specialists, particularly cardiologists.^10,11^

**Figure 1:** Office blood pressure and home blood pressure monitoring targets in 2019 and 2020; BP: blood pressure; HBPM: home blood pressure monitoring; chi-square test.

As compared with 2019, in 2020, there was an increase in the control rates for office BP < 130/80 mmHg (27.2% vs. 30.2%; p<0.02) and HBPM < 130/80mmHg (42.4% vs. 47.5%; p<0.0001) (Figure 1). The SPRINT^13^ (Systolic Blood Pressure Intervention Trial) demonstrated that targeting lower systolic blood pressure than the standard target resulted in higher CV protection, which has been considered by the guidelines.

It is worth pointing out that the COVID-19 pandemic that started in 2020, could have negatively impacted the BP control rates, and yet, an increase in the control rates was observed instead. A recent Brazilian study with more than 50,000 individuals did not find any influence of the pandemic on BP control rates, determined by office BP or HBPM.^14^

Elderly patients usually show greater difficulty in controlling their BP,^1,2,12^ and in the present study an increase in BP control rates by office BP <130/80mmHg and HBPM was observed in older patients. Studies with older hypertensive patients have emphasized the benefits of greater reductions in BP on CV protection.^15,16^ Also, obesity is a condition that has a large impact on BP,^1,2^ and we found an increase in BP control rates from 2019 to 2020, by both office BP and HBPM. These data reinforce the importance of evaluating BP by both methods.^1,2,5,6^

In the total sample, the distribution of hypertension significantly changed from 2019 to 2020, with increases in the rates of CH and WCUH, and reductions in MUH and UH (Figure 2). Therefore, the percentage distribution of the phenotypes improved from one year to the next, even adopting more strict cut-off criteria for HBPM. In addition, the phenotype distribution revealed higher rates of MUH and lower rates of WCUH than those estimated by the 2020 Brazilian Guidelines on Hypertension^1^ and those reported in a Brazilian study with 6,500 patients,^10^ which may be explained by the use of a lower cut-off point for HBPM.^17,18^

**Figure 2:** Distribution of hypertension phenotypes in 2019 and 2020; CH: controlled hypertension; WCUH: white coat uncontrolled hypertension; MUH: masked uncontrolled hypertension; UH: uncontrolled hypertension; chi-square test, *p < 0.05.

Some limitations should be noted: 1) the analysis of two cross sectional cohort of hypertensive patients precludes the evaluation of treatment course; 2) more detailed clinical data of the patients are not known, including the stage of hypertension, the presence of comorbidities and other CV risk factors; 3) data on medication use were available (and incomplete) in less than half of patients. On the other hand, one strength of this study was the sample size, with relatively homogeneous cohorts in 2019 and 2020 for most of demographic and clinical features evaluated.

In conclusion, the data from this study revealed an increase in BP control rates using both office BP <130/80mmHg and HBPM in treated hypertensive patients. In 2019, HBPM was implemented to be used more frequently, and in a regular manner. This may have influenced the practice of physicians, towards greater attention to the measurement of BP levels out of the office, with a consequent increase in the rates of BP control from 2019 to 2020. In addition, HBPM has improved patient engagement in treatment, and has been associated with higher compliance and better blood pressure control.^19,20^ Altogether, these data demonstrate the important contribution of HBPM in increasing the rates of BP control.

## *Supplemental Materials

For additional information, please, click here
